# Study protocol for a randomized controlled trial of RealConsent2.0: a web-based intervention to promote prosocial alcohol-involved bystander behavior in young men

**DOI:** 10.1186/s13063-023-07797-w

**Published:** 2023-12-12

**Authors:** Laura F. Salazar, Dominic J. Parrott, David DiLillo, Sarah Gervais, Anne Marie Schipani-McLaughlin, Ruschelle Leone, Kevin Swartout, Lauren Simpson, Renita Moore, Tiffany Wilson, Nyla Flowers, Haley Church, Amanda Baildon

**Affiliations:** 1 School of Public Health, Georgia State University, P.O. Box 3995, Atlanta, GA 30302-3995 USA; 2 Department of Psychology, Georgia State University, P.O. Box 5010, Atlanta, GA 30302-5010 USA; 3 Department of Psychology, University of Nebraska, Lincoln, 238 Burnett Hall, Lincoln, NE 68588-0308 USA

**Keywords:** Randomized controlled trial, Alcohol use, Bystander behavior, Sexual violence, Virtual reality

## Abstract

**Background:**

Sexual violence (SV) is a significant, global public health problem, particularly among young adults. Promising interventions exist, including prosocial bystander intervention programs that train bystanders to intervene in situations at-risk for SV. However, these programs suffer from critical weaknesses: (1) they do not address the proximal effect of alcohol use on bystander decision-making and (2) they rely on self-report measures to evaluate outcomes. To overcome these limitations, we integrate new content specific to *alcohol use within the context of prosocial bystander intervention* into an existing, evidence-based program, *RealConsent1.0*. The resulting program, *RealConsent2.0*, aims to facilitate bystander behavior among sober *and* intoxicated bystanders and uses a virtual reality (VR) environment to assess bystander behavior in the context of acute alcohol use.

**Methods:**

This protocol paper presents the design of a randomized controlled trial (RCT) in which we evaluate *RealConsent2.0* for efficacy in increasing alcohol- and non-alcohol-involved bystander behavior compared to *RealConsent1.0* or to an attention-control program (“*Taking Charge*”). The RCT is being implemented in Atlanta, GA, and Lincoln, NE. Participants will be 605, healthy men aged 21–25 years recruited through social media, community-based flyers, and university email lists. Eligible participants who provide informed consent and complete the baseline survey, which includes self-reported bystander behavior, are then randomized to one of six conditions: *RealConsent2.*0/alcohol, *RealConsent2.0*/placebo, *RealConsent1.0*/alcohol, *RealConsent1.0*/placebo, *Taking Charge*/alcohol, or *Taking Charge*/placebo. After completing their assigned program, participants complete a laboratory session in which they consume an alcohol (target BrAC: .08%) or placebo beverage and then engage in the Bystanders in Sexual Assault Virtual Environments (BSAVE), a virtual house party comprising situations in which participants have opportunities to intervene. Self-reported bystander behavior across alcohol and non-alcohol contexts is also assessed at 6- and 12-months post-intervention. Secondary outcomes include attitudes toward, outcome expectancies for, and self-efficacy for bystander behavior via self-report.

**Discussion:**

*RealConsent2.0* is the first web-based intervention for young men that encourages and teaches skills to engage in prosocial bystander behavior to prevent SV while intoxicated. This is also the first study to assess the proximal effect of alcohol on bystander behavior via a VR environment.

**Trial registration:**

Clinicaltrials.gov, NCT04912492. Registered on 05 February 2021

## Administrative information

Note: the numbers in curly brackets in this protocol refer to SPIRIT checklist item numbers. The order of the items has been modified to group similar items (see http://www.equator-network.org/reporting-guidelines/spirit-2013-statement-defining-standard-protocol-items-for-clinical-trials/).

**Table:** 

Title {1}	Study protocol for a randomized controlled trial of RealConsent2.0: A web-based intervention to promote prosocial alcohol-involved bystander behavior in young men
Trial registration {2a and 2b}.	Clinicaltrials.gov, NCT04912492. Registered on 05 February 2021.
Protocol version {3}	This is the first version of the protocol (January 02, 2019).
Funding {4}	Research reported in this publication was supported by the National Institute on Alcohol Abuse and Alcoholism of the National Institutes of Health under award number [5R01AA027517].
Author details {5a}	Laura F. Salazar, Georgia State University, School of Public Health Dominic J. Parrott, Georgia State University, Department of Psychology David DiLillo, University of Nebraska, Department of Psychology Sarah Gervais, University of Nebraska, Department of Psychology Anne Marie Schipani-McLaughlin, Georgia State University, School of Public Health Ruschelle Leone, Georgia State University, School of Public Health Kevin Swartout, Georgia State University, Department of Psychology Lauren Simpson, University of Nebraska, Department of Psychology Renita Moore, Georgia State University, Department of Psychology Tiffany Wilson, Georgia State University, School of Public Health Nyla Flowers, Georgia State University, School of Public Health Haley Church, University of Nebraska, Department of Psychology Amanda Baildon, University of Nebraska, Department of Psychology
Name and contact information for the trial sponsor {5b}	Michael A. Mathisen, CRA Associate Director Office of Sponsored Programs 404-413-3523 817-233-6935 cell mmathisen@gsu.edu
Role of sponsor {5c}	The study sponsor and study funders do not have any role or ultimate authority in the study design; collection, management, analysis, and interpretation of data; writing of the report; or in the decision to submit the report for publication.

## Introduction

### Background and rationale {6a}

Sexual violence (SV) is a major problem in the USA and globally [1–5] and exists on a continuum from “minor” behaviors (e.g., catcalling, verbal suggestions of intent of forced sex) to extreme behaviors such as attempted or completed rape [6]. One in four women and one in 26 men have experienced completed rape in the USA [7]. Both male and female young adults aged 18–24 experience the highest SV victimization across any age group [1, 5, 8–10]. Moreover, more than 80% of female and male victims of SV report experiencing victimization before age 25. However, despite decades of research, rates of SV victimization have not decreased [3, 11]. The consistently high incidence of SV is likely due to a major gap in the availability of effective SV prevention programming. To close this gap, critical weaknesses in the extant literature upon which these programs are based must be addressed. One key weakness, and the focus of the present project, involves the way in which SV prevention programming addresses alcohol use.

It is well-established that alcohol is a key contributor to SV, as research indicates that about 50–77% of SV incidents involve alcohol use by the victim, perpetrator, or both [12, 13]. Further, SV often occurs at or after attending bars or parties where attendees drink alcohol [14–16], and approximately 50–80% of men endorse perpetrating unwanted physical contact (e.g., pressing up against a woman from behind, grabbing a woman’s butt) in a bar or party setting [17]. Importantly, in addition to perpetrators and victims of SV, third-party “bystanders” are oftentimes consuming alcohol in situations in which SV is most likely to occur [18]. The bystander decision-making model, which encourages individuals to step in and intervene to prevent SV, is used extensively and effectively in SV prevention [2, 6, 19–25]. However, these prevention programs do not explicitly focus on alcohol use as a potential barrier to successful bystander invention. This is problematic because theory and research indicate that alcohol intoxication functions as a barrier to intervention across all steps of the decision-making process, thereby reducing the likelihood of bystander intervention [26–29]. Recent findings indicate that alcohol use is cross-sectionally associated with lower rates of bystander intervention, especially among men [18, 28, 30]. Although there have been studies that have examined the proximal effect of alcohol consumption on bystander intervention behaviors [30–39], only two of these [30, 37] have *used controlled alcohol administration methods* and assessed *observable bystander behavior* (e.g., verbalizations or actions to prevent SV).

Various SV programs have used the bystander model to increase bystander intervention [40]. One of these programs is *RealConsent1.0*, a web-based SV prevention and bystander intervention program that has been rigorously tested and found to be effective in preventing SV perpetration and increasing prosocial bystander behaviors among college men [23]. *RealConsent1.0 and other e*xisting programs address the role of alcohol in SV [23, 41, 42]; however, our review of the literature indicates that these programs do not integrate alcohol-specific content with the aim of (1) educating bystanders about how alcohol use functions as a barrier to intervention and (2) teaching strategies to compensate for these effects. We present a protocol for a definitive, multi-site RCT to address these limitations. The protocol has been extensively peer-reviewed as part of the funding application process.

## Objectives {7}

The overarching goal of this project is to evaluate the efficacy of *RealConsent2.0*, which includes new program segments for promoting prosocial bystander behavior among intoxicated men. This aim is grounded in the evidence-based premise [28, 43, 44] that integrating alcohol-specific content within the context of *RealConsent1.0* (i.e., “*RealConsent2.0*”) will enhance prosocial bystander behavior among intoxicated bystanders. As such, we sought to achieve this goal by testing two related research questions: (1) Does *RealConsent2.0* facilitate more prosocial bystander intervention (assessed within a virtual environment) among intoxicated men relative to two comparison conditions (i.e., *RealConsent1.0* and *Taking Charge*)? and (2) Does *RealConsent2.0* facilitate more prosocial bystander intervention (assessed at 6- and 12-month follow-up) involving alcohol-related events relative to the two comparison conditions?

## Trial design {8}

**Fig. 1:**
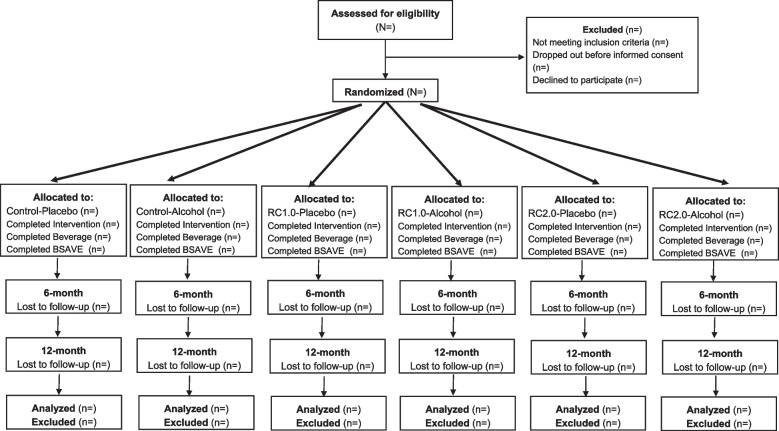
CONSORT 2010 Flow Diagram

This trial is a 2 × 3 factorial, double-blind (i.e., participants and statistician), multisite, superiority randomized controlled trial with six parallel groups (see Fig. 1). The two independent variables are the web-based health promotion intervention (*RealConsent1.0*, *RealConsent2.0*, or *Taking Charge*) and beverage condition (Alcohol or Placebo). Eligible participants (40% of whom will be heavy-episodic drinkers) who provide informed consent will be randomized to one of six conditions using stratified, block randomization with equal allocation across groups. The primary study outcome is bystander decision-making and will be assessed via observable behaviors within a VR environment and via self-report within an online survey administered at baseline and 6- and 12-month post-intervention. This trial will include detailed, written instructions to achieve uniformity of the performance procedures, including screening, retention efforts, and adherence to CONSORT guidelines.

## Methods: Participants, interventions and outcomes

### Study setting {9}

The study is being implemented at two large, public universities in the USA: Georgia State University (Atlanta, GA) and University of Nebraska–Lincoln (Lincoln, NE). Recruiting from these two distinct geographical regions that vary in ethnic and racial diversity should maximize generalization of findings.

### Eligibility criteria {10}

#### Participant inclusion criteria

- Age: 21–25 years, inclusive
- Gender: identifies as a man or transgender man
- Alcohol: must have consumed weight-based amount of alcohol (e.g., five drinks for weight of 160–189 lbs.) at least three times in the last year
- Language: able to read and write English fluently

#### Participant exclusion criteria

- Weight: over 250 lbs.
- Relationship status: in a relationship over 6 months; married/living together
- Medications/conditions: a condition or medication use in which alcohol consumption is medically contraindicated
- Neurological disorders: diagnosis of a neurological disorder
- Psychiatric disorders: past or current diagnosis of bipolar disorder (I or II), schizophrenia, schizoaffective disorder, or any other psychotic disorder
- Treatment for alcohol or drug use: currently being treated for alcohol or drug problems; currently interested in seeking treatment for drinking or drug use
- Physical disability: any physical disability that would prevent an individual from participating in the virtual reality task (self-reported and determined)
- Significant hearing problems: significant hearing problems that would prevent an individual from hearing and responding to the virtual reality task (self-reported and determined)
- Cardiac pacemaker
- Asthma: emergency room visit related to asthma in the past year; use of inhaler more frequently when drinking; use of oral steroid treatments for asthma in the past year
- Legal: any legal restrictions against drinking (e.g., as a condition of probation or parole)
- Alcohol abstinence: individuals who consume alcohol monthly or less; individuals who have consumed the amount of alcohol they would be expected to drink during the lab session (determined by their weight) less than three times in the last year
- Head injury: any past serious head injuries (as indicated by HELPS Brain Injury Screening Tool) [45]
- Acute psychiatric symptomatology: elevated psychological distress as indicated by a score greater than 65 on the Brief Symptom Inventory (BSI) [46]

### Who will take informed consent? {26a}

Trained research staff will obtain informed consent via Zoom during the baseline session. Research staff take each potential participant through the informed consent process and provide opportunities for questions. Participants who agree to participate are asked to sign the informed consent form electronically.

### Additional consent provisions for collection and use of participant data and biological specimens {26b}

N/A

We do not have additional consent provisions for collection and use of participant data. We are not collecting biological specimens as part of this trial.

## Interventions

### Explanation for the choice of comparators {6b}

The Pragmatic Model for Comparator Selection in Health-Related Behavior Trials was used to select an optimal comparator intervention [47]. The main objectives of this trial are to determine whether (1) *RealConsent2.0* is efficacious in increasing alcohol-involved and non-alcohol involved bystander behavior and (2) observed effects in *RealConsent2.0* are greater than the original *RealConsent1.0*. The first objective requires a comparator, such as the use of an attention control that can isolate certain intervention components or underlying mechanisms of behavior change [47]. Accordingly, an Internet-based program called *Taking Charge* was selected as the comparator intervention to serve as the attention control*. Taking Charge* was chosen as the attention-matched control, as it mimics *RealConsent2.0* in several ways, but does not overlap in content. *Taking Charge* is of similar length and access is through a browser-based site but optimized for the smartphone environment. It is interactive, dynamic, and engaging, and it includes multi-media components such as video and graphic presentations imbedded within the context of general health promotion. *Taking Charge* encompasses five modules; however, one module, “Alcohol and Health” was not used in this trial as the content overlaps with *RealConsent*2.0 and could potentially affect study outcomes. The following *Taking Charge* modules were used: (1) My Health Profile—individual assessment of health behaviors with personalized feedback; (2) Staying Healthy—includes information on healthy eating, physical activity, and self-care; (3) Managing Stress—information on identifying stressors and cognitive-behavioral techniques for reducing stress, and (4) Resources/Tools for Goal Setting and Tracking Progress.

### Intervention description {11a}

The original *RealConsent1.0* is a web-based educational program that has two primary goals: (1) to prevent SV perpetration and (2) to increase bystander intervention for SV. These goals are achieved by affecting theoretically and empirically derived mediators, such as increasing knowledge of and skills for safely intervening, correcting misperceptions in normative beliefs, affecting negative attitudes toward date rape, increasing knowledge of effective consent, affecting masculine gender roles, enhancing communication skills, and increasing empathy for victims [23, 48, 49]. *RealConsent1.0* is delivered via a password-protected Web portal that allows participants to access the program either via the web or their mobile phones. The program consists of six 30-min modules with each ranging in number of segments (1–14) and types of activities. Each module involves interactivity, didactic activities, and entertainment-education [50, 51] in the form of 12 mini-episodes of a serial drama called “Crew,” which allows for modeling of positive behaviors and illustration of both positive and negative outcome expectations for perpetrating SV and for bystander intervention.

*RealConsent2.0* incorporates newly developed content into the different modules of *RealConsent1.0* with the objective of increasing knowledge of how alcohol impairs cognitive processing, in particular attentional processes (see Alcohol Myopia Theory [52]), and thus affects their ability: (1) to notice a SV event, (2) to identify events as high risk for SV, (3) to take responsibility to intervene, and (4) to formulate a plan (see integrative framework for the proximal effect of alcohol on bystander intervention for SV [28]. *RealConsent2.0* also attempts to focus men’s attention on salient peer and social norms (see Integrated Model of sexual assault and acquaintance rape [41]) and encourages them to apply good decision-making strategies that can be used while drinking alcohol, enables them to identify alcohol-related risky situations for a sexual assault, helps build new skills to enhance prosocial intervening while drinking alcohol, and develops a positive self-evaluation attached to SV intervention while consuming alcohol or within alcohol contexts.

Following the completion of the baseline survey, participants are randomized to either *Taking Charge, RealConsent1.0*, or to *RealConsent2.0* and are subsequently provided access to their respective programs via email. Each intervention is self-paced, and participants are encouraged to complete their respective programs within 2 weeks.

#### Alcohol administration

At baseline, participants are randomly assigned to either consume an alcohol beverage (told alcohol, receive alcohol) or a placebo beverage (told alcohol, receive no alcohol) in a subsequent laboratory session. Alcohol participants are administered two drinks consisting of an overall dose of 0.80 g/kg body weight of 95% ethanol USP mixed in a 1:5 ratio with orange juice. The beverage is poured into two glasses in equal quantities. This single alcohol dose reliably produces BrACs between .08% and .12% within 20–40 min of beverage consumption. Placebo participants are administered an isovolemic beverage consisting of 5 parts orange juice and 1 part tonic water divided equally into two glasses. Each glass of the placebo beverage is mixed with 4 c.c.s. of 95% ethanol USP and surface layered with an additional 4 c.c.s. of 95% ethanol USP. The rims of the glasses are also sprayed with alcohol.

Regardless of beverage condition, all participants are told that they are receiving a “moderate” dose of alcohol. Twenty minutes are allotted for beverage consumption. Drinks are administered at equally spaced times during the 20-min interval to control for drinking rate. BrACs for participants are monitored every 5 min after finishing their beverages with the Alco-Sensor FST breath analyzer (Intoximeters, Inc., St. Louis, MO). Alcohol participants begin the B-SAVE task after reaching .08% on the ascending limb of the BrAC curve. Placebo participants begin the B-SAVE task immediately after they finish their drinks because placebo manipulations are most effective shortly after beverage consumption [53, 54].

To verify the success of the placebo manipulation, the Subjective Intoxication Scale [55] is administered before and after beverage consumption. Participants are asked to rate their current level of intoxication on a scale from 0 (not at all drunk) to 11 (more drunk than I have ever been). An estimate of how many standard drinks participants believe they consumed is also assessed. A successful placebo manipulation is evidenced by participants’ reporting they ingested “some” alcohol and felt “somewhat” intoxicated [56]. Any participant who denies consuming alcohol or provides a subjective intoxication rating of “0” is excluded from analysis.

#### Criteria for discontinuing or modifying allocated interventions {11b}

By participant request only.

#### Strategies to improve adherence to interventions {11c}

Participants are encouraged to complete their assigned intervention within 2 weeks. Study staff send a total of three reminder emails (one email every 4 days during the 2-week period). In addition, we provide a $10 incentive to complete an acceptability survey following completion of each intervention module.

#### Relevant concomitant care permitted or prohibited during the trial {11d}

Participating in an alcohol or drug treatment program is an exclusion criterion, but we do not monitor or prohibit participation during the trial.

#### Provisions for post-trial care {30}

It is the policy of each affiliated institution to not pay for post-trial care; however, mental health and counseling services are available.

#### Outcomes {12}

**Fig. 2:**
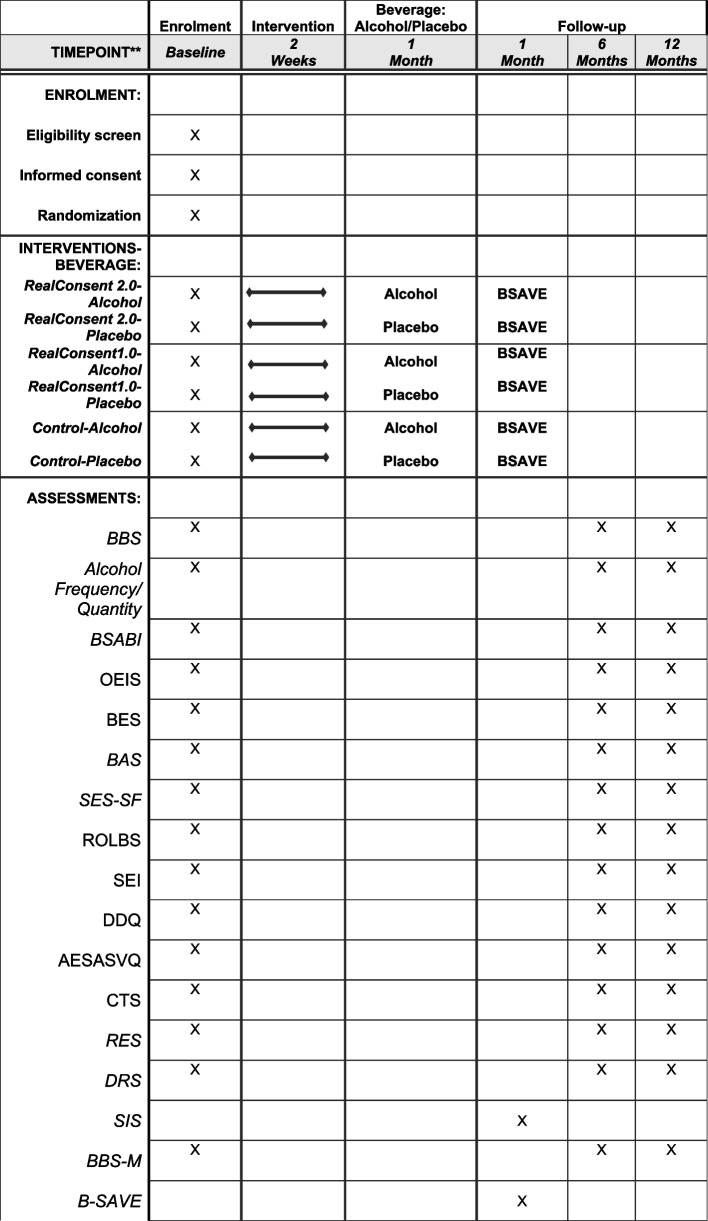
SPIRIT diagram of trial schedule of enrolment, intervention, and assessments. Note: BSAVE (Bystanders in Sexual Assault Virtual Environments), BBS (Bystander Behavior Scale), BSABI (Barriers to Sexual Assault Bystander Intervention); OEIS (Outcome Expectancies for Intervening Scale); BES (Bystander Efficacy Scale); BAS (Bystander Attitudes Scale); SES-SF (Sexual Experience Survey Short Form); ROLBS (Reactions to Offensive Language & Behavior Scale); SEI (Self-Efficacy to Intervene Scale); DDQ (Daily Drinking Questionnaire); AESASVQ (Alcohol Expectancies Regarding Sex, Aggression, and Sexual Vulnerability Questionnaire); CTS (Revised Conflict Tactics Scale); RES (Rape Empathy Scale); DRS (Differential Reinforcement Scale)

The SPIRIT diagram is displayed in Fig 2. The primary outcome measures are bystander behavior using the B-SAVE [57] 1-month post-intervention and self-reported bystander behavior during the past 6 months measured using the 44-item Bystander Behavior Scale (BBS) [58] at baseline and 6- and 12-month post-intervention.

Within each of the five at-risk scenarios depicted in the BSAVE, participants are prompted at two points (with a flashing microphone icon) to verbalize their response in that situation—thus providing ten opportunities to intervene in sexual risk situations. A team of research assistants will use audio recordings of participants’ open-ended responses to the risk scenes, as well as non-verbal behaviors captured by the VR system, to code intervention behaviors in response to each risk scenario. The most central code of interest will reflect the presence or absence of intervention attempts (0 = no, 1 = yes) in response to each of the two SV intervention opportunities within each of five risk scenes. An index measure will be created based on these responses that can range from 0 to 10 and will be used in data analyses.

For each item on the BBS, participants are asked to report their engagement with a specific bystander behavior (i.e., engaged in behavior, did not engage in behavior, no opportunity to engage in behavior). Following completion of the 44 items, follow-up questions are administered to assess additional information relevant to each bystander behavior in which the participant reportedly engaged or did not engage. For behaviors in which the participant reportedly engaged, the follow-up questions assess the number of times participants engaged in the behavior on a 1 (1 time) to 3 (3 or more times) scale. Questions also prompt participants to recall the most recent time they engaged in the behavior and report whether (1) they were drinking alcohol, (2) others were drinking alcohol, and (3) the people involved were friends or strangers. For behaviors in which the participant reportedly did not engage, follow-up questions assess the number of times they had the opportunity to intervene but did not do so using a 1 (1 time) to 3 (3 or more times) scale. Questions also prompt participants to recall the most recent time they did not engage in the behavior (but had an opportunity to do so) and report whether (1) they were drinking alcohol, (2) others were drinking alcohol, and (3) the people involved were friends or strangers.

#### Secondary outcomes

The following secondary measures will be assessed.

1. Barriers to sexual assault bystander intervention as measured using the 16-item *Barriers to Sexual Assault Bystander Intervention Scale* [59]. Each item is rated on a 1 (strongly disagree) to 7 (strongly agree) scale, with higher mean scores indicating more barriers to intervention.
2. Outcome expectancies for intervening as measured using the 17-item *Outcome Expectancies for Intervening Scale* [60]. Each item is rated on a 1 (strongly disagree) to 7 (strongly agree) scale, with higher mean scores indicating greater positive expectancies for intervening.
3. Bystander efficacy as measured using the 14-item *Bystander Efficacy Scale* [60]. Response options for each item range from 0 to 100% confidence. A score is created by subtracting the mean of these 14 items from 100 to create a scale of perceived efficacy, with higher scores reflecting greater bystander efficacy.
4. Bystander attitudes as measured using the 12-item *Bystander Attitudes Scale* [61]. Each item is rated on a 1 (minimum) to 5 (maximum) scale, with higher mean scores indicating higher intentions of intervening.
5. Sexual violence perpetration as measured using the 35-item *Sexual Experience Survey Short Form Perpetration* [62]. Participants indicate the number of times that they perpetrated SV using verbal coercion, incapacitation, threats of physical force, and physical force on a scale ranging from 0 (never) to 3 (3 or more times). The mutually exclusive scoring system will be used [63]. The goal is to count people only once according to the most severe act perpetrated.

#### Tertiary outcomes and other measures

1. Alcohol use quantity and frequency as well as heavy episodic drinking was measured by *NIAAA’s* Alcohol Use Disorder and Associated Disabilities Interview Schedule-5 (AUDADIS-5) [64].
2. Self and descriptive peer drinking norms as measured by the *Daily Drinking Questionnaire* [65]. Participants report how much alcohol they consume and how much alcohol they believe their peers consume during an average week over the past 3 weeks. Drinks per week are summed.
3. Alcohol expectancies for aggression, sexual affect, sexual drive, and vulnerability to sexual coercion as measured by the *Alcohol Expectancies Regarding Sex, Aggression, and Sexual Vulnerability Questionnaire* [66]. Each item is rated on a 1 (not at all) to 5 (very much) scale. Scores are obtained by summing items on each subscale (aggression, sexual affect, sexual drive, and vulnerability) and dividing by the number of items on that scale. Higher means scores on each subscale reflect higher alcohol expectancies.
4. Sexual violence perpetration in dating relationships as measured by the 7-item sexual coercion subscale of the *Revised Conflict Tactics Scale* [67]. Participants indicate the number of times that they perpetrated on a 7-point scale ranging from 0 (never) to 6 (more than 20 times). A frequency score is calculated by adding the midpoints of responses for each item, with higher scores reflecting greater sexual violence perpetration in dating relationships in the past year.
5. Rape empathy as measured by the *Rape Empathy Scale* [68], which is comprised of 18-items that assess rape-victim empathy and 18-items that assess rape-perpetrator empathy on a 1 (strongly disagree) to 5 (strongly agree) scale. A mean score is computed for each subscale, with higher scores reflecting greater empathy.
6. Peer disapproval for sexual aggression as measured by the 3-item *Differential Reinforcement Scale* [69]. Participants rate their level of agreement for each statement on a 1 (very approving) to 5 (very disapproving) scale, with greater mean scores indicating greater disapproval for sexual aggression.
7. Peer network density as measured by the *Peer Density Network scale* [70]. Participants are asked to list five male peers with whom they most often associated in high school as well as the strength of each relationship between pairs on a 0 (never met) to 100 (extremely close friends) scale. Network density is calculated by averaging relationship strength across each of these peer relationships.

#### Participant timeline {13}

##### Sample size {14}

Sample size requirements were determined via a Monte Carlo simulation study based on Aim 2 analysis strategy, which will require a larger sample than Aim 1 analyses. The primary outcome for Aim 2 will be intervention proportion: the number of times each participant engaged in prosocial bystander behavior divided by the number of sexist or violent situations they encountered during a given assessment period. Sample size is therefore based on the number per treatment group needed to detect clinically meaningful effects on intervention proportion. Calculations were based on 80% power, level of significance of .05, and two-tailed statistical tests. Clinically meaningful treatment efficacy was based on previous research [23, 71] and was defined as having between a small and medium effect size between group intervention proportions (Cohen’s *h* ≥ .30). In this RCT, this effect size translated into a 15% point difference between groups, at a minimum, and could potentially translate to a clinically significant increase in bystander intervention for those who receive *RealConsent2.0*. Assuming control participants intervene in approximately 50% of all sexist or violent *non-alcohol* situations they encounter, those who receive RealConsent1.0 will intervene in 65% of those non-alcohol-related situations; those who receive RealConsent2.0 will also intervene in 65% of non-alcohol-related situations. For *alcohol-related* situations, we expect those in control and in RealConsent1.0 to intervene approximately 30% of the time whereas for RealConsent2.0, we expect 45% of the time they will intervene. Based on these proportion differences, a total of 484 participants are needed ~161 in each intervention group). We expect between 20% attrition based on previous work. To achieve adequate power, we estimated a needed sample size of at least 605 to maintain complete data for 484 participants for the per protocol analysis. Additionally, this sample size allows us to detect small differences (7%) in intervention rates within the VR paradigm as a function of intervention and beverage conditions, as well as their interactions.

#### Recruitment {15}

Participants are recruited from metropolitan areas of Atlanta, Georgia, and Lincoln, Nebraska, using in-person and online recruitment strategies. In-person methods include fliers posted throughout the community (e.g., grocery stores, coffee shops, university campuses), direct recruitment (research staff handing out fliers at public venues), and snowball sampling (at the end of the lab session, participants are given a flier and encouraged to give it to someone who may be interested). Online methods include social media advertisements (e.g., Instagram, Facebook, TikTok, Twitter), advertisements on other websites (e.g., Craigslist), and university email listservs.

Both sites meet monthly to discuss the number of participants enrolled in the past month and set goals for the number of participants to be enrolled in the upcoming month. Each site meets weekly to discuss recruitment in relation to that month’s enrollment goal. Each site makes independent decisions to adjust recruitment accordingly by posting new community fliers, increasing the social media ad budget, increasing the number of participants emailed through university listservs, and/or employing other approved recruitment strategies that are not currently being used at that site. The project coordinators from each site meet bi-weekly to discuss current enrollment numbers and recruitment strategies utilized at each site.

A practical aspect of our study was that it was funded in September of 2019 and was substantially affected by the COVID-19 pandemic. In 2020 and 2021, many restaurants and bars were still closed or were operating at limited capacity with local mask mandates. As this study involves assessing alcohol consumption specifically within contexts involving groups of people socializing and drinking, we delayed the start of recruitment to ensure our results would be valid. Also, a significant part of our protocol involves in-person assessments where participants are scheduled to come into our labs located on each participating campus for the alcohol administration and the VR assessment. Thus, even with academic campuses opening up, we surmised that recruitment would be difficult given that participants would need to feel safe coming into our building, interacting with people, and taking off their mask to consume alcohol. Once restrictions were lifted and more restaurants and bars opened up in early 2022, we made the decision to begin recruitment. At first, recruitment efforts were slow; however, since January 2023, we have seen an increase in recruitment and have set an anticipated end date for recruitment of our baseline sample.

## Assignment of interventions: allocation

### Sequence generation {16a}

A separate allocation sequence was created for each site using an automated *stratified block randomization* program implemented through SAS. Participants are stratified by drinking level, resulting in two strata: heavy episodic drinkers (HED) and non-HED. For each stratum, a separate block randomization sequence was used to ensure that equal numbers of men are assigned across the six groups that represent treatment condition (*RealConsent1.0, RealConsent2.0, or Taking Charge)* by beverage condition (Alcohol or Placebo): *(1) RealConsent2.0*/alcohol, (2) *RealConsent2.0*/placebo, (3) *RealConsent1.0*/alcohol, (4) *RealConsent1.0*/placebo, (5) *Taking Charge*/alcohol, or (6) *Taking Charge*/placebo.

### Concealment mechanism {16b}

The prepopulated allocation spreadsheets are stored on a secured, password protected Dropbox folder for each site. Personnel involved in outcome assessment {i.e., the BSAVE coders and study statistician) do not have access to these spreadsheets.

### Implementation {16c}

The allocation sequence was generated by the project statistician and stored in the form of two separate prepopulated randomization spreadsheets, one for each site. Each spreadsheet contains two tabs, one for participants who meet the criterion for heavy episodic drinking and one for participants who do not. Once a new participant completes the Baseline Survey and passes the required data validity checks, the project coordinator checks responses to determine whether or not the participant meets the criterion for heavy episodic drinking. The project coordinator then selects the appropriate tab in the randomization spreadsheet and assigns the next available condition to the participant. The participant’s condition is then recorded on study tracking spreadsheets, so that research staff who are responsible for contacting participants and running study sessions know to which intervention and beverage condition the participant is assigned.

## Assignment of interventions: Blinding

### Who will be blinded {17a}

Participants are blinded to the main study hypotheses. They are informed that they will be randomized to view one of three different web-based health promotion programs and that each program covers topics related to young men’s physical and mental health and well-being as well as how to deal with stressful situations. In addition, the biostatistician and outcome assessors, who code the B-SAVE data, are blinded to intervention and beverage condition.

### Procedure for unblinding if needed {17b}

We are implementing three interventions during this trial, all of which are educational in nature and designed to affect behavior. There are no circumstances under which unblinding is permissible.

## Data collection and management

### Plans for assessment and collection of outcomes {18a}

Primary, secondary, and tertiary outcome data (see “Outcomes”) are measured using reliable and valid instruments and collected via Qualtrics survey platform at baseline as well as at 6- and 12-months following the completion of the web-based intervention. To promote data quality, several “attention” questions (e.g., “fill in ‘strongly agree” for this question”) are embedded within the Qualtrics survey and are checked by study staff. In addition, even though participants are told that they are allowed to “skip” individual questions, they are also told that they must complete 75% of questions overall and 1005 of the bystander behavior questions to receive compensation. The primary outcome of bystander behavior is also measured during the laboratory session via the B-SAVE VR environment. The BSAVE is a reliable and valid measure of bystander behavior [72].

### Plans to promote participant retention and complete follow-up {18b}

Contact information for participants, including phone numbers, mailing addresses, secondary email addresses, social media handles, and the phone and email of an alternate contact are collected at the time of enrollment to support follow-up efforts. Participants indicate their preferred mode of contact at that time as well. Retention is also prompted by use of an online system (Calendly), which allows participants to schedule their baseline and lab sessions independently by choosing from a variety of openings at different days and times. A text or email reminder is sent to participants 24 h prior to their baseline and lab sessions, with more frequent reminders sent prior to the 6- and 12- month follow-up surveys. During the lab session and after completing their 6-month follow-up survey, participants are asked to review and update their contact information. The incentive structure is also expected to support participant retention in this study. Participants receive $25 for completing the baseline session, $10 per module of the intervention program completed ($40–60 total) plus a $20 bonus if they complete the program within 1-week, $10 per hour for completing the lab session ($30–80 total), $40 for completing the 6-month follow up survey, and $50 for completing the 12-month follow up survey.

### Data management {19}

Two forms of data are being collected during this trial. Survey data collected via Qualtrics survey platform and B-SAVE audio recordings. Following completion of each survey via Qualtrics, study staff examine data validity (i.e., “attention check” items) and completeness. Survey data are stored on Qualtrics servers, which are protected by high-end firewall systems. Data are encrypted using Transport Layer Security (TLS) encryption (also known as HTTPS) for all transmitted data that are downloaded onto password-protected computers by the study PIs. Final datasets will be password-protected. Data management procedures are performed by the study staff.

The second form of data are the audio recordings of B-SAVE responses. Audio files are recorded on computer that operates the B-SAVE. After each laboratory session, these files are uploaded to a password-protected folder on a secure server. B-SAVE recordings are then transcribed and any identifying information is removed. B-SAVE transcripts are then double coded by a team of highly trained coders to ensure reliability.

### Confidentiality {27}

Confidentiality of participants will be protected by assigning participants a unique study identification number (USIN) that will be connected to study data. No personal information will be connected to study data. There will be restricted access to any personal information; personal information will be stored on password protected computers and secure servers separate from data. Only certified study personnel will have access to personal information. Following completion of the study, all identifying information will be destroyed.

### Plans for collection, laboratory evaluation, and storage of biological specimens for genetic or molecular analysis in this trial/future use {33}

N/A. This trial does not include collection of biological specimens.

## Statistical methods

### Statistical methods for primary and secondary outcomes {20a}

Data collected within the virtual environment will be structured as the proportional count of intervention behaviors relative to the number of opportunities to intervene. We will test all Aim 1 hypotheses using generalized linear models based on either Poisson or negative binomial distributions, depending on if a given model is overdispersed. These are appropriate statistical models to use in this case because we expect this proportional count outcome to be positively skewed based on pilot research. We will analyze *Aim 2* RCT data with an intent-to-treat and per protocol analyses. An intent-to-treat analysis includes all participants who were randomized, regardless of compliance, withdrawal, and anything that happens after randomization. An advantage is that it is an analysis based on original randomization; however, effect estimation may be conservative and misleading with increasing attrition. A per protocol analysis considers only participants who fully complied and completed the study. Per protocol is less conservative and may reflect true treatment differences for those with full compliance. Including an intent-to-treat and per protocol analysis will provide a more complete understanding of treatment effects. Outcomes, including prosocial bystander behaviors, will be modeled using a generalized linear mixed model. This is an appropriate statistical model to use with repeated measures data collected over time [73]. Random effects for subject will be included to account for the multiple measurements taken at baseline and 6- and 12-month follow-up on each subject. Intervention condition will be dummy coded, with variables representing both the *RealConsent1.0* and *RealConsent2.0* conditions, with *Taking Charge* as reference group. Planned contrasts will be conducted between *RealConsent1.0* and *RealConsent2.0* groups.

### Interim analyses {21b}

We do not plan to conduct interim analyses related to any study aims. It is expected that the trial will be terminated upon reaching a target sample size, which affords sufficient power to test the study aims. It is possible, though highly unlikely, that the trial would be terminated due to concerns about the safety and/or welfare of human subjects. In this case, PIs Salazar and Parrott will make the final decision to terminate the trial in consultation with the Data Safety Monitoring Board.

### Methods for additional analyses (e.g., subgroup analyses) {20b}

N/A. This trial does not include prespecified subgroup analyses.

### Methods in analysis to handle protocol non-adherence and any statistical methods to handle missing data {20c}

Because we will most likely encounter incomplete data due to dropouts and non-response, multiple imputation of missing data will be used to impute missing values based on other available covariates.

### Plans to give access to the full protocol, participant level-data, and statistical code {31c}

Drs. Salazar and Parrott at Georgia State University will field all requests for the full protocol, data, and statistical code and will ensure that data are made available, when applicable. Access to these data will be provided through the Inter-University Consortium for Political and Social Research (ICPSR). Users will be required to agree to the conditions of use governing access to the public release data, including restrictions against attempting to identify study participants, destruction of the data after analyses are completed, reporting responsibilities, restrictions on redistribution of the data to third parties, and proper acknowledgment of the data source and funders. Within 1 year after the study is completed, all data will be compiled and organized into a single repository at ICPSR. Data will be made publicly available through ICPSR's website. We estimate that this process will be completed no later than 2026.

## Oversight and monitoring

### Composition of the coordinating center and trial steering committee {5d}

Dr. Salazar and Dr. Parrott oversee the standardization of procedures across investigators and staff, encompassing both sites. At each site, a dedicated project coordinator supervises project personnel, including temporary student research assistants and support staff, while also facilitating the organization of team project meetings. Dr. Parrott serves as the primary director of communication for the multisite research team. He ensures that effective communication among study investigators and staff is achieved through various means, including email correspondence, regular weekly site meetings, and monthly team meetings. Recruitment endeavors are regularly monitored by the respective project coordinators at each site. The trial employs online recruitment methods, which involve the use of social media advertisements, outreach initiatives, and targeted email communications. Prospective participants undergo an initial screening process using an online screener, and those meeting eligibility criteria can proceed to schedule a virtual interview for further eligibility assessment. During these eligibility verification sessions, trained staff including student research assistants assess and confirm eligibility status before obtaining informed consent from participants.

### Composition of the data monitoring committee, its role, and reporting structure {21a}

This trial includes a Data Safety Monitoring Board (DSMB) and Data and Safety Monitoring Plan. The DSMB members were chosen by Drs. Salazar and Parrott. The three voting members were chosen based upon their knowledge of clinical trial methodology, experience with the topical area (i.e., sexual violence risk reduction strategies), and absence of conflicts of interest. They have been appointed for the life of the project.

DSMB meetings are held every 12 months and began the first year of recruitment. Serious adverse events will be reported to the Chair as soon as they occur. The Chair of the DSMB determines whether an in-person meeting or teleconference is needed. Prior to the meetings, a written report containing any study preliminary findings is sent to DSMB members. Preliminary findings are not made available to individuals outside of the DSMB. Each meeting includes time to review the progress of the study and to answer questions from members of the DSMB. Members of the DSMB disclose any potential conflicts of interest, either pre-existing or those that develop during their tenure, to the Principal Investigator and the NIH Project Officer.

In accordance with NIH policy, a data and safety monitoring plan was developed for the proposed study. Co-PIs Salazar and Parrott provide oversight of all recruitment and study procedures. They also oversee weekly quality assurance checks during recruitment and assessment time periods to ensure recruitment goals and validity of the data. All records pertaining to the study and all of the original and electronic files containing collected data are securely stored. Drs. Salazar and Parrott are jointly responsible for dissemination of study findings through presentation and publication formats.

The Institutional Review Board at University of Nebraska Lincoln (UNL) serves as the single IRB or record and approved all Informed Consent documents for the study and provides additional oversight of data and safety issues. The study protocol was approved prior to soliciting or consenting any participants. Moreover, the study is reviewed on an annual basis by the UNL IRB with regard to recruitment and retention, and annual reports will be made by the Co-PIs to the DSMB Chair regarding the progress of the proposed project, including any issues pertinent to recruitment, retention, confidentiality, and safety of human subjects. Any incidents that involve a breach of this plan or serious accident/injury will be reported to the DSMB Chair, the IRB chair at UNL, and the NIAAA Safety and Monitoring Board.

The members of the DSMB perform the following activities:

1. Review the research protocol and plans for data and safety monitoring.
2. Review progress of the trial, including analysis of data quality and timeliness; subject recruitment and retention; subject risk versus benefit; and other factors that may affect outcome.
3. Review serious adverse event reports, provide commentary, and provide oversight to ensure that reports are relayed to individual IRBs and to the Office of Human Research Protections (OHRP), as indicated.
4. Review analyses of outcome data and review reports of related studies to determine whether the current study needs to be changed or terminated.
5. Determine whether the trial should continue as designed, should be changed, or should be terminated based on the data and make recommendations to the NIH and the Institutional Review Board considering conclusion or continuation of the study.
6. Protect the confidentiality of the trial data and the results of the monitoring.
7. Determine whether and to whom outcome results should be released prior to the reporting of study results.
8. Following DSMB meetings, provide appropriate NIH staff with written information concerning their findings.

### Adverse event reporting and harms {22}

In the case of an adverse event (AE), a written report will be prepared for submission to the IRB Chair at the University of Nebraska-Lincoln (UNL) within 48 h. In the event of a serious adverse event (SAE), the same procedures will be followed and, in addition, the SAE will be reported within 48 h to the Data Safety Monitoring Board (DSMB) Chair and NIAAA Program Official. The report of AEs or SAEs will include whether they were anticipated or unanticipated, a brief narrative summary of the event, a rating of severity of the event, a description of the impact on participants, the total number of participants impacted by the event, whether enrolled participants should be notified of the event, and a determination of whether a causal relationship existed between the study procedures and the event. Reports will also contain actions taken to prevent recurrence as well as whether the protocol and/or informed consent document should be changed as a result of the event. The UNL IRB will determine whether it is appropriate to stop the study protocol temporarily or will provide suggestions/modifications to the study procedures as necessary. Finally, as part of the annual progress report to the DSMB and NIAAA, summary information regarding all AEs and SAEs occurring during that year will be provided.

### Frequency and plans for auditing trial conduct {23}

Dr. Salazar and Dr. Parrott, both Multiple Principal Investigators (MPIs) at the GSU site, collaborate with Dr. DiLillo and Dr. Gervais, Co-Investigators (Co-Is) at the UNL site, to oversee the rigorous quality control and reliability of various trial procedures. These procedures encompass screening, baseline assessments, randomization, the alcohol administration protocol, and follow-up assessments. To ensure seamless execution, members of the study team at each site provide daily task completion reports and promptly direct any issues or eligibility-related questions to the respective site's Principal Investigator (PI) or Co-I. Furthermore, weekly team meetings are held at each site, focusing on critical aspects such as recruitment objectives, eligibility protocols, adherence to the study's protocol, and the meticulous scrutiny of data quality. The recruitment process is regularly monitored on a weekly basis at each site. Additionally, both sites engage in monthly team meetings to collectively uphold the implementation of relevant Institutional Review Board (IRB) policies and ensure the correct execution of study procedures. These meetings also serve to evaluate progress toward recruitment objectives and introduce supplementary recruitment strategies if necessary.

### Plans for communicating important protocol amendments to relevant parties (e.g., trial participants, ethical committees) {25}

This is a multi-site trial with UNL acting as the single IRB (sIRB) of record. As such, UNL is the central site responsible for submitting any modifications across sites to be approved by the UNL Institutional Review Board (IRB). Proposed changes to the protocol impacting study operations are first agreed upon by MPIs and Co-Is prior to being submitted as a formal amendment through UNL’s IRB platform. Each modification is listed as a numbered amendment on a document shared with key personnel at GSU to garner feedback prior to submission and ensure a centralized record of changes. GSU key personnel are notified once each amendment is submitted and approved. MPIs and Co-Is communicate regularly via regular monthly meetings to ensure appropriate implementation of those changes. If the protocol changes impact active participants who need to be notified of the changes, both sites agree on an action plan for reaching out to identified participants. ClinicalTrials.gov is updated as needed at the time IRB amendments are approved.

## Dissemination plans {31a}

The Co-Principal investigators for this NIH-funded clinical trial will adhere to the National Institutes of Health (NIH) Policy on Dissemination of NIH-funded Clinical Trial Information. Georgia State University (GSU), the recipient institution for this award, has an internal policy in place that ensures all clinical trials are registered and results reporting are publicly posted. This policy can be found in GSU’s institutional review board policy.

In PY01, we registered the proposed trial at ClinicalTrials.gov *prior* to enrollment of participants, which complied with the requirement for registration to be submitted within 21 days after the first human subject is enrolled. We included a specific statement in our study’s informed consent documents relating to the posting of clinical trial information at ClinicalTrials.gov. Following the completion of the trial and data analyses, we will submit summary results for public posting. This timeframe complies with NIH policy, as results must be posted no later than 1 year after the study's Primary Completion Date, as described in 42 CFR 11.44(a) of the final rule.

## Discussion

Much of the research on alcohol and SV prevention has focused on reducing alcohol misuse, heavy drinking, and perpetration. Such efforts are justified and needed; however, limited attention has been given to integrating alternative behaviors that are more positive, healthy, and prosocial into prevention programs or to evaluating the potential impact of such content on the outcomes of prevention programs [13]. One such strategy that is considered a positive and prosocial approach is the bystander education model. Although there are many bystander behavior programs that have been implemented and tested for efficacy in affecting bystander behavior [21, 22, 24, 32, 40, 71], to our knowledge, this paper describes a protocol for the first randomized controlled trial to test the efficacy of a novel, web-based intervention designed to increase *alcohol-involved bystander behavior*. This protocol also includes a description of methods for testing the proximal effects of alcohol use on *actual* bystander behavior within a VR environment. Given that alcohol use is present among 50–77% of SV incidents [13], the most significant contribution of the proposed project will be to provide the first evidence of how to effectively promote prosocial bystander behavior in men who have consumed alcohol and improve future development, evaluation, and dissemination of bystander intervention programs. In doing so, this project will provide the necessary empirical foundation for existing bystander prevention programming to translate these findings directly into real-world applications that will ultimately have a significant impact on high rates of SV that have been persistent on college campuses.

There are some limitations in this study. One of the primary objectives is to determine the efficacy of RealConsent2.0 in increasing alcohol-involved bystander behavior in comparison to the original RealConsent1.0 and to an attention comparator web-based program. Although this study involves an RCT, which is the gold standard for research designs and controls for many threats to internal validity, we are unable to control for potential external events that may influence study outcomes. In addition, our trial is being conducted in two distinct geographic areas that may limit the generalizability of our results. Nonetheless, there are some strengths. Mainly that with our use of a VR environment, our design will allow us to determine the proximal effect of alcohol use on *actual* bystander behavior. Also, our 12-month follow-up period will allow us to determine if any effects observed are sustained over time.

## Trial status

This is the first version of the protocol (January 02, 2019). Recruitment began on March 1, 2022. The completion of baseline recruitment (*N* = 605) is expected in August 2024.

## Abbreviations

SV: Sexual violence
VR: Virtual reality
